# Evaluation of gene xpert for routine diagnosis of HIV-associated tuberculosis in Nigeria: A prospective cohort study

**DOI:** 10.1186/s12890-017-0430-6

**Published:** 2017-05-30

**Authors:** Maxwell Oluwole Akanbi, Chad Achenbach, Babafemi Taiwo, John Idoko, Agatha Ani, Yetunde Isa, Oche Agbaji, Christiana Ukoli, Patrick Akande, Mamoudou Maiga, Robert Leo Murphy

**Affiliations:** 1 Institute of Public Health and Medicine, Northwestern University, Chicago, IL USA; 2 Department of Medicine, University of Jos, Jos, Nigeria; 3 Department of Medicine, Northwestern University, Chicago, IL USA; 4 Department of Medical Microbiology, University of Jos, Jos, PL Nigeria; 5 AIDS Prevention Initiative in Nigeria (APIN) Center, Jos University Teaching Hospital, Jos, PL Nigeria; 6 AIDS Prevention Initiative in Nigeria (APIN), Abuja, Nigeria; 7 Department of Biomedical Engineering, Northwestern University, Evanston, IL USA

**Keywords:** Xpert, Tuberculosis, HIV, Nigeria, Africa, Outcomes research

## Abstract

**Background:**

Xpert MTB/Rif (Xpert) is described as a game changer in tuberculosis (TB) control. We evaluated the impact of Xpert on diagnosis, time to treatment, and treatment outcome among patients with HIV associated TB in Nigeria.

**Methods:**

Adults with HIV being evaluated for pulmonary TB (PTB) were consecutively enrolled into the study cohort. At baseline, expectorated sputa were examined using Xpert and smear microscopy for *Mycobacterium tuberculosis* (MTB) and acid fast bacilli, respectively. Patients diagnosed with TB were followed-up until 6 months post TB diagnosis. TB was defined as sputum positive by smear microscopy, Xpert detection of MTB (bacteriologically confirmed case), or clinician diagnosed TB with initiation of full TB treatment (clinical diagnosis). Time to treatment was time from first clinic presentation for TB evaluation to initiation of TB treatment. We examined the proportion PTB patients with a positive Xpert result and compared time to TB treatment and outcome of TB treatment in patients based on sputum test results.

**Results:**

A total of 310 adults with HIV were enrolled. The median CD4 cell count was 242 (interquartile range (IQR) 120–425) cells/mm^3^ and 88.1% were receiving antiretroviral therapy (ART). PTB was diagnosed in 76 (24.5%) patients, with 71 (93.4%) being bacteriologically confirmed. Among patients with PTB, 56 (73.7%) were Xpert positive. Median time to treatment was 5 (IQR 2–8) days and 12 (IQR 5–35) days in patient with and without Xpert positive results, respectively; *p* = 0.005. Overall 73.1% had symptom free survival at 6 months post PTB treatment initiation with no significant differences observed based on TB test method. 10 (14.9%) died within 6 months of TB treatment initiation. In analysis adjusted for age, sex, and mode of diagnosis (Xpert positive or negative), only ART use independently predicted mortality (AOR 0.10; 95% CI 0.01–0.93).

**Conclusion:**

The use of Xpert for routine care reduced time to PTB treatment, but did not improve survival in patients with HIV treated for susceptible PTB.

## Background

Despite antiretroviral therapy (ART), tuberculosis (TB) remains a major cause of morbidity and mortality among persons with human immunodeficiency virus (HIV) infection in Sub-Saharan Africa [1]. Prompt and accurate diagnosis of TB and timely initiation of appropriate treatment decreases TB transmission and mortality [2]. To aid prompt TB diagnosis, the World Health Organization (WHO) in 2010 endorsed the Cepheid Xpert® MTB/RIF (Xpert) as a first line tool for diagnosis of HIV-associated TB [3]. Xpert is a nucleic acid amplification test that simultaneously detects MTB and rifampicin resistance, and has demonstrated high sensitivity (79.7–100%) as well as shorter diagnostic turnaround time (<2 h) when compared to TB culture [4–6]. As at 2015, 60% of countries recommended Xpert as the initial TB test for persons with possible drug-resistant (DR) TB, and 69% recommended it as the initial diagnostic test in cases of presumptive HIV-associated TB [7].

Nigeria, a country with high TB burden, commenced roll-out of Xpert in 2012 [8]. Xpert roll-out has however been slow, and sputum smear microscopy (SM) for acid fast bacilli (AFB) remains the primary mode of diagnosing TB in Nigeria. SM has a sensitivity of about 35% in children and in persons with HIV-infection [9, 10]; populations most adversely affected by TB in sub-Saharan Africa [11]. In recognition of these challenges, the government of Nigeria has prioritized Xpert use for people with HIV, suspected DR-TB, and children. Since introduction of Xpert in Nigeria, the few available studies have focused on its diagnostic performance in non-HIV clinical settings [12, 13]. Understanding the role of Xpert in the diagnosis of HIV-associated TB, as well as its impact on treatment outcomes, is crucial given the high mortality associated with this condition.

Using prospectively collected data during routine clinic care, we evaluated the impact of Xpert on diagnosis, time to treatment, and outcome of HIV-associated pulmonary tuberculosis (PTB). Our primary hypothesis was that 70 (±10) % of patients treated for PTB will have a positive Xpert result. Our secondary hypothesis was that PTB patients with positive Xpert result will initiate treatment earlier than those diagnosed using SM or clinical diagnosis. Lastly we explored the impact of Xpert on outcome of PTB treatment.

## Methods

### Study design and participants

The Jos University Teaching Hospital (JUTH) HIV clinic acquired a four module Xpert machine in 2012, and by 2013 Xpert became the standard first-line tool for evaluation of HIV-associated PTB. We enrolled consecutive HIV positive adults (≥18 years), accessing care at the JUTH HIV clinic referred for PTB testing using Xpert between June 2013 and June 2015 into the study cohort. We excluded known TB patients being evaluated for DR-TB. Written informed consent was obtained from all study participants and the study was approved by the Ethics Review Boards of Northwestern University, Chicago, USA and Jos University Teaching Hospital, Nigeria.

### Study setting

The JUTH HIV treatment program commenced with support from the Government of Nigeria and the Bill & Melinda Gates Foundation in 2002. Subsequent support from the United States President’s Emergency Plan for AIDS Relief (PEPFAR) in 2004 expanded access to free antiretroviral therapy (ART). At the time of this study approximately 10,000 patients were receiving comprehensive HIV care, which included treatment of opportunistic infections such as TB.

### HIV treatment and monitoring

HIV treatment was guided by the Nigerian National ART guidelines [14]. Patients with a CD4 count of <350 cells/mm^3^, and those with TB, pregnancy, or Hepatitis B irrespective of CD4 count were commenced on ART. Routine follow-up of patients on ART occurred monthly or every other month for drug pick-up, adherence counseling, and targeted clinical assessment. HIV treatment response was monitored using CD4 T-cell count and HIV-1 RNA assayed on site with Partec CyFlow Counter and Roche PCR Amplicor Monitor version 1.5, respectively. These tests were performed at presentation, ART initiation, and every 6 months subsequently for CD4 T-cell count and yearly for HIV-1 RNA assay.

### TB diagnosis and treatment

In line with the WHO TB treatment guideline adopted by Nigeria [15], all HIV positive individuals were screened for TB symptoms at all clinic encounters. Patients with symptoms suggestive of PTB (presumptive PTB cases) were referred to the in-house TB Directly Observed Treatment (DOT) center, where sputa were collected for SM and Xpert testing at the JUTH TB Laboratory. Two expectorated same-day sputa samples were obtained from each patient for testing. SM was carried out on un-concentrated sputum samples as previously described [16]. One Xpert test was carried out using combined samples from each patient and was reported as: (1) MTB detected rifampicin resistance detected, (2) MTB detected rifampicin resistance not detected, (3) MTB not detected, or (4) Indeterminate. Patients with indeterminate results had the test repeated using fresh samples, and results of the repeat Xpert test were documented as the final Xpert result. External quality control for SM was carried out by the National TB Control program, while quality control for Xpert was carried out using the manufacturer’s manual [17].

The JUTH TB laboratory was the only facility with Xpert capability in the state at the time of the study and received samples from other hospitals. In addition to the Xpert, the GenoType MTBDRplus assay [18], another molecular diagnostic tool, was available but mostly used for research purposes. JUTH also housed a free-standing regional TB laboratory that carried out TB culture using solid media, which was routinely done for patients with rifampicin resistance following Xpert as per protocol. Chest X-ray facilities were also available and were requested at the discretion of the treating physician.

Based on the WHO case definition, a patient was classified as having PTB if sputum sample was positive by SM or Xpert (bacteriologically confirmed case) or if SM and Xpert were negative but the treating physician made a diagnosis of PTB and initiated full TB treatment (clinical diagnosis) [15]. TB cases, without rifampicin resistance on Xpert were treated with quadruple anti-tubercular therapy for six months consisting of rifampicin (or rifabutin, if on a protease inhibitor), isoniazid, ethambutol, and pyrazinamide for the first two months, then rifampicin and isoniazid for the remaining four months. Patients with rifampicin resistant PTB were referred to designated DR-TB treatment facilities for specialized care.

The in-house TB DOT center routinely documented date of initiation of TB treatment and treatment outcome in a designated register. The register was provided and supervised by the Nigerian National TB control program. Based on WHO guidelines [15], TB treatment outcome was reported as:

1. Cured: a PTB patient with bacteriologically confirmed TB at the beginning of treatment who was smear- or culture-negative in the last month of treatment and on at least one previous occasion.
2. Completed treatment: a TB patient who completed treatment without evidence of failure but had no record to show that sputum smear or culture results in the last month of treatment, and on at least one previous occasion were negative, either because tests were not done or because results are unavailable.
3. Treatment failure: a TB patient whose sputum smear or culture was positive at month 5 or later during treatment.
4. Default: a TB patient who whose treatment was interrupted for 2 consecutive months or more
5. Death: a TB patient who died for any reason during the course of treatment.

The sum of cured and treatment completed was reported as treatment success.

### Data collection

During routine clinic visits, patient data were collected by health providers and transferred to an electronic database (File Maker Pro). Data from the laboratory (hematology, chemistry, and CD4 T-cell count) and pharmacy (medication history, pick-up dates, and percentage adherence) were entered on the same database and merged, to aid real time patient management.

At study baseline (day of first presentation for evaluation for presumptive PTB), in addition to routine sputum collection for SM and Xpert test, a structured interviewer-administered questionnaire was used to obtain demographic data, patient symptoms, and history of previous TB treatment. This questionnaire was updated at all patients’ subsequent visits to the clinic, to capture date of TB diagnosis, date of initiation of TB treatment, and outcome of TB treatment. For patients without a diagnosis of TB, follow-up was terminated when a diagnosis of TB was excluded. For patients diagnosed with TB without rifampicin resistance, follow-up continued until outcome of TB treatment was determined. If Xpert indicated rifampicin resistance, follow-up terminated at point of diagnosis because patients were referred to the specialized DR-TB program. We extracted CD4 T cell counts (pre-ART and most recent-within 3 months of study baseline interview) from the electronic patients’ records.

### Variables

Patients with either bacteriologically confirmed PTB or clinical PTB were grouped together as PTB [15]. Time to treatment was computed as the number of days from the date of baseline visit to the date of initiation of TB treatment. The outcome of PTB treatment was documented at the end of the 6^th^ month of TB treatment and corroborated using the DOTS TB register at the JUTH HIV clinic. For patients who did not complete 6 months of treatment, follow-up was terminated on the date of death (which is usually reported to the clinic and captured in the electronic patient records) or date of last clinic visit (loss to follow-up).

The main exposure was Xpert result indicating ‘MTB detected’. In addition, PTB diagnosis was further categorized as: (1) both Xpert and SM positive (Xpert+/SM+), (2) positive Xpert and negative SM (Xpert+/SM-), (3) Xpert negative and SM positive (Xpert-/SM+), and (4) negative or indeterminate Xpert and negative SM (Xpert-/SM-). Other variables of interest were age, sex, number of TB symptoms, CD4 T-cell count at TB diagnosis, use of ART at time of TB diagnosis, and history of prior TB treatment.

### Statistical analysis

The study population was characterized using descriptive statistics and the data of patients with and without PTB were compared using chi square test, t-test, or Mann Whitney U tests as appropriate. The number of patients with PTB with positive Xpert or SM results was presented as proportions, and compared using chi square. Time to treatment was compared using Kaplan Meier analysis. The outcome of TB treatment was reported as a proportion. Predictors of treatment outcomes were determined using logistic regression models. In all regression models, factors were included in the multivariate model if they had a *p* < 0.2 in the univariate model or had been shown to predict outcome in previous studies. A *p* < 0.05 was considered statistically significant.

Statistical analyses were done using Stata version 14 (College Station, TX).

## Results

**Fig. 1:**
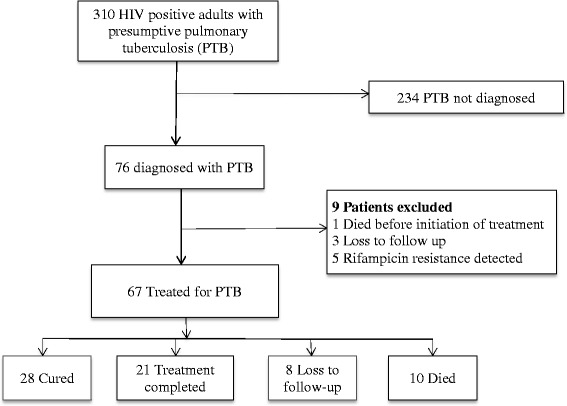
Study Flow diagram showing patient enrolment, tuberculosis treatment and outcomes

A total of 310 HIV-positive adults were enrolled onto the study cohort. Of these, 195 were females (62.9%). The mean age (standard deviation (SD)) was 38.5 (10.6) years. Males were older than females; 41.6 (10.6) years, 36.6 (10.7) years, *p* = 0.0001. The median CD4 cell count at time of TB testing was 242 (Interquartile range (IQR) 120–425) cells/mm^3^, and 88.1% of the participants were on ART. The study flow chart is shown in Fig. 1.

### Tuberculosis diagnosis

A total of 76 (24.5%) patients had a diagnosis of PTB. Among PTB patients, 56 (73.7%) had MTB detected by Xpert, 18 (23.7%) had no MTB detected, and 2(2.6%) had persistent indeterminate results after a repeat Xpert. 5/56 (8.9%) had rifampicin resistance. Of the patients diagnosed with PTB, 57 (75%) were SM positive. Sixty three patients (82.9%) had bacteriologically confirmed PTB. Further analysis showed that 50 patients (65.8%) had Xpert+/SM+, 6 (7.9%) Xpert+/SM-, 7 (9.2) Xpert-/SM+, and 13 (17.1%) Xpert-/SM- sputum test results. Table 1 shows patients characteristics stratified by PTB diagnosis. Patients treated for PTB who had a positive Xpert results (irrespective of SM) were more likely to be male, report weight loss or night sweats, and have positive sputum SM results (Table 2).

**Table 1:** Characteristics of 310 adults with HIV evaluated for pulmonary tuberculosis, Nigeria (June 2013- June 2015)

Characteristics	Total Population (*n* = 310)	Treated for TB (*n* = 76)	No evidence of TB (*n* = 234)	*P*-value
Age,years, Mean (SD)	38.5 (10.6)	35.7 (9.6)	39.4 (10.7)	0.007
Sex				0.03
Male, n (%)	115 (37.1)	36 (47.4)	79 (33.8)	
Female, n (%)	195 (62.9)	40 (52.6)	155 (66.2)	
Body Mass Index	21.9 (3.9)	20.9 (3.8)	22.3 (3.9)	0.02
TB symptoms				
†Cough duration,Weeks, Median (IQR)	3 (2–6)	4 (2–8)	3 (2–5)	0.002
Fever, n (%)	227 (73.2)	62 (81.6)	165 (70.5)	0.06
Weight loss, n (%)	250 (80.6)	67 (88.2)	183 (78.2)	0.06
Night Sweat, n (%)	209 (67.4)	56 (73.7)	153 (65.4)	0.18
Cough > 2 weeks, n (%)	195 (62.9)	56 (73.7)	139 (59.4)	0.03
Previous TB treatment, n (%)	81 (26.1)	11 (14.5)	70 (29.9)	0.008
On ART, n (%)	273 (88.1)	63 (82.9)	210 (89.1)	0.34
†Latest CD4 Count, cells/mm^3^, (Median, IQR)	242 (120–425)	224 (99–354)	259 (128–457)	0.11

*SD* Standard deviation, *IQR* Interquartile range, *TB* Tuberculosis, *ART* Antiretroviral Therapy, †Mann Whitney U test

**Table 2:** Characteristic of 76 adults with HIV treated for pulmonary tuberculosis, Jos, Nigeria (June 2013– June 2015)

Characteristics	Xpert MTB Detected (*n* = 56)	Xpert No MTB Detected (*n* = 20)	*P*-value
Age, years,Mean (SD)	37.3 (13.2)	35.0 (12.3)	0.39
Sex			0.004
Male, n (%)	32 (57.1)	4 (20.0)	
Female, n (%)	24 (42.9)	16 (80)	
TB symptoms			
Cough > 2 weeks, n (%)	42 (75.0)	14 (70.0)	0.66
Fever, n (%)	48 (85.7)	14 (70.0)	0.12
Weight loss, n (%)	52 (92.9)	15 (75.0)	0.03
Night Sweat, n (%)	45 (80.4)	11 (55)	0.03
Previous TB treatment, n (%)	7 (12.5)	4 (20)	0.4
On ART, n (%)	48 (88.9)	15 (83.3)	0.54
Current CD4 ≤ 200 cells/mm^3^,n (%)	38 (67.9)	10 (50.0)	0.16
^a^CD4 count at TB diagnosis, cells/mm^3^, Median (IQR)	198 (68–354)	309 (123–507)	0.09
SM positive, n (%)	50 (89.3)	7 (35.0)	<0.001
Rifampicin Resistance, n (%)	5 (8.93)	-	-

*SD* Standard deviation, *IQR* Interquartile range, *TB* Tuberculosis, *MTB* Mycobacterium tuberculosis, *ART* Antiretroviral Therapy, *SM* Sputum smear microscopy for acid fast bacilli ^a^Kruskal Wallis test

### Time to treatment

**Fig. 2:**
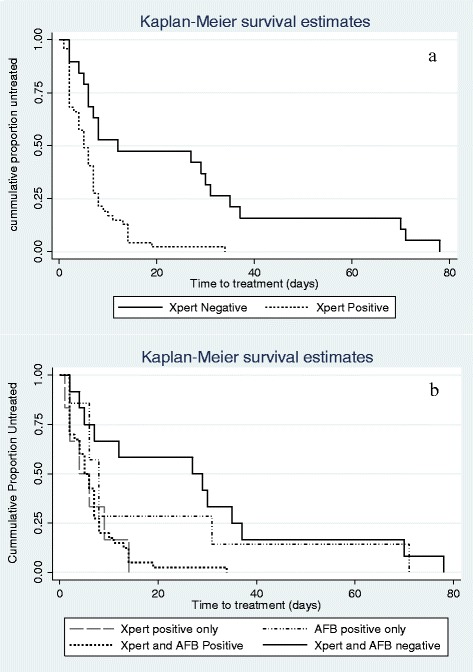
Kaplan Meier curves of time to pulmonary tuberculosis treatment among 67 adults with HIV in Jos, Nigeria. Legend for fig. 2: Fig. 2a compares time to treatment in adults with HIV-associated tuberculosis with positive and negative Xpert. Time to treatment was significantly shorter in patients with positive Xpert results. In (**b**), four patient subgroups are defined using a combination of Xpert and Acid fast bacilli (AFB) result on smear microscopy. Similar to (**a**), Xpert positive adults with HIV-associated tuberculosis had shorter time to tuberculosis treatment, while the greatest treatment delay was in those with negative Xpert & AFB results

Time to initiation of TB treatment was analyzed in 67 patients who initiated TB treatment. The median time to treatment was 6 (IQR 2–12) days. Only three patients (4.2%) commenced TB treatment on the day of baseline visit. Cumulatively 47.2% commenced treatment within 5 days of baseline visit. Median time to treatment was 5 (IQR 2–8) days and 12 (IQR 5–35) in patient with and without Xpert positive results respectively; Mann Whitney U *p* = 0.0045. The median time to treatment was 5 (IQR 2–7) days for Xpert+/SM+, 6 (IQR 4–9) days for Xpert+/SM-, 8 (IQR 6–31) days for Xpert-/SM+, and 27 (IQR 5–35) days for Xpert-/SM- cases; Kruskal Wallis *p* = 0.04. Figure 2 shows differences in time to treatment based on TB sputum test results. Using Kaplan-Meier analysis, time to treatment was shortest in patients with Xpert+/SM+ and longest in Xpert-/SM-test results (Log rank *p* = <0.001 Fig. 2a and *p* = 0.002 Fig. 2b).

### Outcomes of TB treatment

Of the 67 patients who initiated PTB treatment, 49 patients (73.1%) achieved TB treatment success (28 cured, 21 completed treatment). Eight patients (11.9%) defaulted and 10 (14.9%) died within 6 months of initiating TB treatment. There was no association between Xpert result and successful TB treatment (Xpert MTB detected, 37/49 (75.5%) versus MTB not detected or indeterminate, 12/49 (25.5%) X^2^ 1.34; *p* = 0.25). Treatment success was recorded in 5/5 (100%), 32/43 (74.4%), 3/6 (50%) and 9/13 (69.2%) of patients diagnosed with Xpert+/SM -, Xpert+/SM+, Xpert-/SM+, and Xpert-/SM- PTB respectively (Chi square 3.61, *p* = 0.31).

A total of 10 deaths were documented within 6 months of initiation of TB therapy (analysis excluded 8 patients who defaulted). Deaths recorded based on diagnostic group were 0/5 (0%) Xpert+/SM-, 5/37 (13.5%) Xpert+/SM+, 1/4 (25%) Xpert-/SM+, and 4/13 (30.8%) Xpert-/SM- (Chi square 3.3, *p* = 0.35). By analysis in which the summation of death and default were considered as a composite poor treatment outcome, 0/5 (0%) Xpert+/SM-, 11/43 (25.6%) Xpert+/SM+, 3/6 (50%) Xpert-/SM+, and 4/13 (30.8%) Xpert-/SM- had poor treatment outcomes (Chi square 3.6, *p* = 0.31). In univariate logistic regression analysis of age, sex, mode of diagnosis (Xpert positive versus negative or indeterminate) and use of ART (Yes/No), only ART reduced poor treatment outcome (OR 0.16; 95% CI 0.03–1.0; *p* = 0.48).

### Predictors of mortality

Table 3 shows predictors of mortality in 59 patients treated for PTB (8 patients who were lost to follow-up were excluded). An Xpert positive result was not associated with lower mortality in bivariate or multivariate analysis. In analysis adjusted for age, sex, and mode of diagnosis (Xpert positive versus negative or indeterminate) odds for death during treatment were 90% lower in patients on ART at time of TB diagnosis (AOR 0.10; 95% CI 0.01–0.93).

**Table 3:** Logistic regression analysis of predictors of mortality in 59 adults with HIV treated for pulmonary tuberculosis in Jos, Nigeria

	Unadjusted Analysis			Adjusted Analysis		
	OR	95% CI	*P*-value	AOR	95% CI	*P*-value
Xpert positive	0.32	0.08–1.32	0.12	0.67	0.11–4.05	0.66
Age, years	1.03	0.97–1.10	0.19	1.04	0.96–1.12	0.31
BMI < 18.5 Kg/m^2^	1.90	0.41–8.82	0.41	-	-	-
Sex, Female	4.52	0.87–23.50	0.07	5.63	0.69–45.93	0.11
3 or more TB symptoms	0.66	0.11–3.81	0.65	-	-	-
Recent CD4 count <200 cells/mm^3^	0.87	0.21–3.50	0.84	-	-	-
Recent CD4 count (cell/mm^3^)	1.00	0.99–1.00	0.66	-	-	-
On ART	0.11	0.02–0.77	0.03	0.10	0.01–0.93	0.04
Time to treatment (days)	0.99	0.95–1.05	0.87	-	-	-

*OR* Odds ratio, *AOR* Adjusted odds ratio, *CI* Confidence interval, *BMI* Body mass index, *TB* tuberculosis, *ART* Antiretroviral therapy

Variables with *p* < 0.2 in the bivariate analysis were included in the adjusted model

## Discussion

We evaluated the diagnosis, time to treatment, and outcome of HIV-associated PTB in a clinic cohort in Nigeria, following the implementation of Xpert as a first-line tool for TB diagnosis. A quarter of patients enrolled had a diagnosis of PTB. The majority of patients diagnosed with PTB (73.7%) had MTB detected using Xpert. Patients with Xpert positive results had significantly shorter time to treatment, but Xpert had no impact on survival.

The use of Xpert is expected to significantly improve the diagnosis of HIV-associated TB compared to microscopy, due to its increased sensitivity [6, 19–21]. However, we observed that among our cohort of patients with HIV-associated PTB, yield from Xpert was similar to microscopy (73.5% Xpert, 75% microscopy, 65.8% concordance). Similar to what we found, a retrospective analysis of data from 13 regions in Namibia showed that 77.1% of HIV patients with Xpert positive results were also positive by microscopy [22]. With this high level of concordance between Xpert and microscopy it is unlikely that Xpert significantly improved sensitivity of TB diagnosis in these populations. Likewise, in Uganda, Xpert did not significantly increase the number of patients starting TB treatment, compared to microscopy, when routinely collected TB program data were analyzed [23]. In contrast to these, Lawn et al. reported that Xpert increased TB diagnostic yield by 45% compared to microscopy in a clinical trial among ART naïve adults with HIV in South Africa [19]. A Cochrane review of 27 clinical trials also showed that Xpert increased TB detection by 23% (95% CI 15–32) when compared to microscopy [24]. It is unclear why the efficacy of Xpert reported in these trials are not being replicated during routine use in clinical settings. The level of immuno-competence of patients may be a contributing factor. Our patient population, in contrast to those in the Lawn study, was mostly on ART and had a higher CD4 count (242 (IQR 120–242 cells/mm^3^ versus 171 (IQR 102–236) cells/mm^3^). Advanced immunosuppression significantly reduces sensitivity of microscopy and use of Xpert may yield greater diagnostic benefit in patients with immunosuppression. A previous study also reported a high rate of microscopy positive PTB in our clinic population [16].

Another factor that could reduce the diagnostic benefit of Xpert during routine use is empirical TB treatment. In our cohort, 17.1% of patients treated for TB, had negative Xpert and microscopy results. TB diagnosis in these patients was likely made using symptoms and chest radiography (empirical therapy). Empirical therapy is widely used even in facilities where Xpert is available [25, 26]. It has been proposed that widespread empirical TB therapy could reduce the impact of Xpert in improving TB diagnosis at the population level [26]. Calling for cessation of empirical treatment, however is unethical since as many as 14–30% of Xpert negative samples end up being culture positive [24].

Rapid diagnosis of rifampicin resistance, a surrogate marker of multidrug resistance TB, facilitates prompt initiation of DR-TB treatment. Five patients, representing 8.9% of TB diagnosis, had rifampicin resistance and were referred to specialized DR-TB treatment facilities. Prompt diagnosis and treatment of DR-TB would reduce disease spread and could lead to better treatment outcomes. This may be a major benefit of Xpert in populations, such as ours, with high prevalence of DR-TB.

One of the limitations of microscopy is its inability to differentiate MTB from other smear positive organisms, particularly non-tuberculous mycobacteria (NTM). Because Xpert specifically detects MTB, smear positive/Xpert negative samples should prompt further evaluation for NTM. This becomes more important in patients with immunosuppressive conditions like HIV, at high risk for NTM [27]. Diagnosis of NTM remains a challenge in resource limited settings because it requires culture of relevant biological samples [28]. Facilities for culture are scarce in these settings. Seven patients (9.2%) in our cohort of patients treated for PTB had Xpert negative/SM positive results, suggesting these may be cases of NTM, and this group had the worse treatment outcome (50% mortality). A high prevalence of NTM has been reported among patients evaluated for TB in this region of Nigeria. A recent study reported that 15% of individuals with culture positive TB had NTM; this proportion rose to 38% in those with HIV [29]. Although not all patients in whom NTM is isolated require treatment, when treatment is indicated, drugs used to treat NTM differ from those for MTB [30]. Patients with NTM treated with conventional TB drugs are unlikely to respond to this treatment and may have poorer treatment outcomes.

The capacity of Xpert to diagnose TB within two hours suggests that patients with symptoms of TB could get a diagnosis and commence treatment during one clinic visit. With smear microscopy, patients have to make at least one more clinic trip to get test results and commence treatment. Some patients never return for the visit to complete sputum collection or commence treatment [31, 32]. We observed significant differences in time to treatment, ranging from a median of 5 days in Xpert positive/smear positive patients to 27 days in Xpert negative/smear negative patients. While Xpert positive cases had shorter time to treatment, only 3 patients (4.2%) commenced treatment during a single clinic visit, which is the presumed norm if Xpert is used. Cumulatively 47.2% commenced treatment within 5 days of baseline visit. Delay in initiating TB treatment despite implementation of Xpert is not unique to our population [26, 33, 34], and we need to better understand factors contributing to this delay. We observed that the four-cartridge Xpert machine at the study site not only served the clinic but the whole state with a population of 3.2 million with an estimated 40,000 cases of TB/HIV co-infection in 2012 [35]. At full capacity, only 12–16 specimens can be processed in a day using a four-module Xpert machine [36], meaning processing of additional specimen will spill into a second day. While deployment of more Xpert machines may be a viable solution, delay in initiation of treatment has also been observed after making test results available to patients during their first visit [26]. Patient contribution to delays in TB treatment remains a challenge which Xpert may not completely eliminate. In a study of the Zambian TB program, patient factors were found to be the major contributor to TB treatment delay [37].

Few studies have evaluated the impact of Xpert on outcomes of TB treatment. Similar to previous studies [33, 38], Xpert result did not predict mortality. Use of ART however independently reduced mortality by 90%. Some factors may attenuate the impact of Xpert on TB outcomes. The first is a prevailing practice of empiric treatment [39]. Irrespective of TB test results, when suspicion for TB is strong, treating physicians are likely to initiate TB therapy, which is reasonable given the sensitivity of Xpert in persons with HIV. Another reason is that recent studies, including ours, did not consider the treatment benefits for patients diagnosed with rifampicin resistance. Improved outcomes for patients with DR-TB, may be one of the main benefits of Xpert.

This study has limitations. The study was carried out at a single site which may limit its generalizability. TB management at the site however followed WHO guidelines, which is widely used in many low- and middle-income countries with high prevalence of TB and HIV. Another limitation was that cultures were not available to confirm PTB diagnosis. While this may be considered a limitation, the goal of the study was not to determine the test characteristics of Xpert or microscopy. Our aim to evaluate the contribution of Xpert to diagnosis and its impact on treatment of HIV-associated TB, during routine use. Moreover, our study setting is similar to health facilities in other low- and middle-income countries where TB culture is not available and Xpert is being rolled out as the first line for diagnosis of HIV associated TB. One other limitation was that sample size for outcome analysis may be underpowered to identify small effect sizes in subgroup analysis. Our outcome analysis also excluded patients with rifampicin resistance.

## Conclusions

We have demonstrated the utility of Xpert in routine clinical evaluation of patients with HIV-associated TB in a middle-income country with high TB and HIV burden. While Xpert did not significantly increase microbiological confirmation of TB among treated cases or outcome of treatment, it did aid rapid diagnosis of rifampicin resistance and resulted in shorter time to treatment. Larger studies incorporating benefits attributable to early diagnosis and treatment of DR-TB will be needed to fully elucidate the impact of Xpert in TB patients. As Xpert is rolled out, its contribution to TB treatment may be modest, due to delays in TB treatment initiation as well as the wide use of empiric treatment. TB programs need to find innovative ways to aid prompt initiation of treatment in patients with Xpert positive TB. Furthermore, other rapid diagnostic tools are also urgently needed to address the 14–30% of culture positive TB patients with negative Xpert results, as well as cases of NTM. Finally, where resources are constrained, ART needs to be prioritized as it was the only factor we identified that independently improved survival during TB treatment.

## Abbreviations

AFB: Acid fast bacilli
ART: Antiretroviral therapy
DOT: Directly observed treatment
DR-TB: Drug resistant tuberculosis
HIV: Human immunodeficiency virus
IQR: Interquartile range
JUTH: Jos University Teaching Hospital
PEPFAR: The United States President’s Emergency Plan for AIDS Relief
PTB: Pulmonary tuberculosis
SD: Standard deviation
SM: Smear microscopy
TB: Tuberculosis
